# Profiling of Barley, Wheat, and Rye *FPG* and *OGG1* Genes during Grain Germination

**DOI:** 10.3390/ijms241512354

**Published:** 2023-08-02

**Authors:** Sylwia Kowalik, Jolanta Groszyk

**Affiliations:** Plant Breeding and Acclimatization Institute–National Research Institute, Radzików, 05-870 Błonie, Poland; s.kowalik@ihar.edu.pl

**Keywords:** BER, embryos germination, grains germination, 8-oxoguanine DNA glycosylase, formamidopyrimidine DNA glycosylase, cereals

## Abstract

This research is about the profiling of barley (*Hordeum vulgare* L.), wheat (*Triticum aestivum* L.), and rye (*Secale cereale* L.) *FPG* and *OGG1* genes during grain germination. During seed germination, reactive oxygen species accumulate, which leads to DNA damage. In the base excision repair (BER) system, the enzymes formamidopyrimidine DNA glycosylase (FPG) and 8-oxoguanine DNA glycosylase (OGG1), among others, are responsible for repairing such damage. We decided to check how the expression of genes encoding these two enzymes changes in germinating grains. Spring varieties of barley, wheat, and rye from the previous growing season were used in the study. Expression level changes were checked using Real-Time PCR. After analyzing the obtained results, the maximum expression levels of *FPG* and *OGG1* genes during germination were determined for barley, wheat, and rye. The results of the study show differences in expression levels specific to each species. The highest expression was observed at different time points for each of them. There were no differences in the highest expression for *FPG* and *OGG1* within one species. In conclusion, the research provides information on how the level of *FPG* and *OGG1* gene expression changes during the germination process in cereals. This is the first study looking at the expression levels of these two genes in cereals.

## 1. Introduction

Plants require the presence of oxygen to live, which enables the cell to carry out the processes necessary to generate energy and their proper functioning. Unfortunately, oxygen is not always an ally of plants, and sometimes can be harmful to them. Oxygen in the molecular form O^2^ is not toxic to plants. In the ground state, molecular oxygen has two unpaired electrons with parallel spins in two opposite orbits. In addition, it has three energy levels in the magnetic field, hence its name triplet oxygen [1]. Conformational changes in oxygen can cause damage to plant cells. Reactive oxygen species (ROS) can lead to changes in cells, causing mutations, and in the worst case, even their death [2]. ROS can be produced as by-products of cell metabolism, as well as a result of stresses to which plants are exposed, e.g., salinity and drought [3]. ROS are considered to be one of the main causes of loss of seed viability and deterioration of germination [4,5]. During seed storage, loss of viability is associated with the accumulation of DNA strand breaks and chromosome aberrations, confirming the link between reduced germination during seed aging and DNA damage [6]. ROS induces many DNA damages, including the formation of guanine modifications, most often 8-oxoguanine (8-oxoG), the level of which increases significantly during seed aging. This damage is repaired by the base excision repair (BER) system, which is mediated by two enzymes, i.e., FPG (formamidopyrimidine DNA glycosylase) and OGG1 (8-oxoguanine DNA glycosylase), responsible for recognizing and removing oxidative DNA lesions. 

### 1.1. The BER Repair System and Its Role in Reducing Oxidative Damage

One of the major DNA repair pathways in all organisms is base excision repair. The BER prevents the cytotoxic and mutagenic effects of damage, that occurs in the nitrogenous bases of DNA, which makes it important in maintaining the integrity of the genome [7]. Oxidative damage such as 8-oxoguanine (the oxidized form of guanine) is mainly repaired by the BER system [8]. The BER repair mechanism is initiated by DNA glycosylases (specific to the damage), which cut the N-glycosidic bond between the damaged base and deoxyribose to form an apurinic/apyrimidinic (AP) site. Next, an AP endonuclease, or AP lyase, is required to remove the AP site, causing degradation to oligonucleotides by breaking phosphodiester bonds within the DNA chain [8]. The next step of the repair pathway can proceed in two ways, via either a “short” or “long” patch mechanism. The selected mechanism is determined by the type of enzyme involved and the change that occurs. The “short patch” mechanism is generated when one incorrect nucleotide needs to exchange, while the “long patch” is activated when 2–13 nucleotides must be repaired. Both pathways require fragments flanked by 3’-OH and 5’-dRP. The “long patch” repair process involves a polymerase that completes the sequences by pushing out the 5’-dRP flanking fragment along with several nucleotides. The endonuclease then removes the fragment pushed out by the polymerase. In the next step, the completed sequence is connected to the DNA strand by ligase action. On the other hand, 5’-dRP lyase participates in the “short patch” mechanism by removing the flanked site. Then, through the action of DNA polymerase and DNA ligase, the removed AP site is supplemented, and the strands are connected. Unfortunately, the identity of the DNA polymerase involved in this process is not yet fully understood. Researchers suggest that it may be polymerase α, but there is no certainty about it. Further research is needed to provide more information about this subject [9].

### 1.2. Activity of DNA Glycosylases

DNA glycosylases are enzymes that recognize damaged or modified bases in DNA and remove them by cleaving the N-glycosyl bond that links the bases to the 2-deoxyribose monosaccharide [10]. Searching for bases in the DNA chain makes it easy to locate changes that do not significantly distort the overall DNA structure. Each biological species has several different DNA glycosylases [8]. Different types of DNA glycosylases are specialized to find specific types of damage and changes. Four structural superfamilies of DNA glycosylases have been identified in plants, including: DNA alkyladenine glycosylase (AAG), DNA uracil glycosylase (UDG), helix-serpentine-helix (HhH-GPD) and helix-double-turn helix (H2TH) [11]. The AAG superfamily consists of compact single-domain enzymes with an α/β structure and a positively charged DNA-binding surface [10]. They are monofunctional glycoslases that remove alkylated purines. AGG genes have been detected in arabidopsis (*Arabidopsis thaliana* L.), brachypodium (*Brachypodium distachyon* L.), grape (*Vitis vinifera* L.), wheat (*Triticum aestivum* L.), and maize (*Zea mays* L.) [12,13,14,15,16]. The UDG superfamily also consists of monofunctional glycosylases, but specialized in the removal of uracil from DNA [17]. They are proteins consisting of a single domain containing a four-stranded twisted β structure flanked by an α-helix [18]. In plants, the *UDG* gene has been detected in wheat, maize, carrots (*Daucus carota* L.), and onions (*Allium cepa* L.) [19,20,21,22]. The HhH-GPD superfamily has a very different spectrum of activity. The hallmark motif of HhH-GPD is a sequence-independent DNA-binding domain. The proteins consist of four N-terminal and six to seven C-terminal α-helices, connected by a type II β-hairpin [23]. The helix-serpentine-helix is then followed by a GPD loop motif containing glycine-G, proline-P and an aspartic acid-D residue [24]. These enzymes remove damage caused by oxidation or alkylation. This group of enzymes includes DNA 8-oxoguanine glycosylases (OGG), which remove the main products of 8-oxoG purine oxidation. The last superfamily is H2TH, containing bifunctional enzymes that have the ability to cross the sugar-phosphate backbone and are mainly involved in the repair of oxidative damage [24]. This superfamily includes formamidopyrimidine DNA glycosylase (FPG) that recognizes oxidative damage such as 8-oxoG, formamidopyrimidine, spiroiminodihydantoin and guanidinohydantoin [25].

The main aim of the presented study was to identify changes in the expression levels of genes encoding FPG and OGG1 proteins, i.e., *FPG* and *OGG1*, respectively, in germinating grains of three different cereals: barley (*Hordeum vulgare* L.), wheat, and rye (*Secale cereale* L.).

## 2. Results

### 2.1. Expression Profiles of FPG and OGG1 Genes during Germination 

These tests allowed for the determination of *FPG* and *OGG1* expression profiles under germinations of three spring cultivar species, i.e., barley (*Hordeum vulgare* L.), wheat (*Triticum aestivum* L.), and rye (*Secale cereale* L.). Expression profiles of *FPG* and *OGG1* genes were determined using germinated embryos collected at 10-time points, i.e., 0.5 hours (h), 1 h, 3 h, 6 h, 9 h, 12 h, 15 h, 18 h, 21 h, and 24 h, after water treatment. Embryos for total RNA extraction were collected between 10:00 a.m. and 12:00 a.m. due to the elimination of differences, which can be the result of circadian rhythms. 

**Figure 1:**
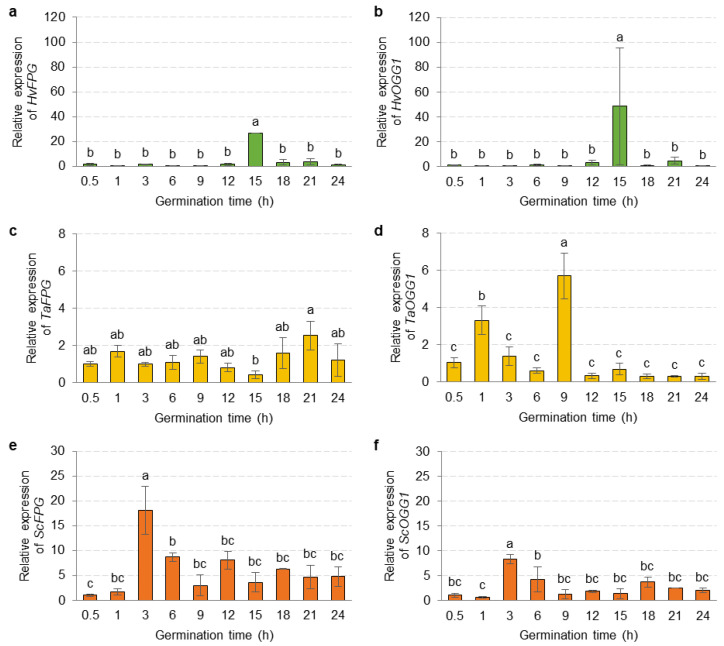
Expression profiles of the genes encoding FPG and OGG1 enzymes of DNA repair pathway in spring barley (**a**,**b**), wheat (**c**,**d**), and rye (**e**,**f**). Grains were germinated in water conditions and embryos for expression analysis of *FPG* (**a**,**c**,**e**) and *OGG1* (**b**,**d**,**f**) genes were collected after 0.5 hours (h), 1 h, 3 h, 6 h, 9 h, 12 h, 15 h, 18 h, 21 h, and 24 h. The results present the mean with standard error (n = 3). Letters indicate significant differences (Table S1), revealed by Duncan’s test.

The Real-Time PCR analysis indicated that *HvFPG* expression was like *HvOGG1* with the highest expression at 15 h (Figure 1a,b). In the early stages of germination between 0.5 h and 12 h, *HvFPG* expression was in the range of 0.18 to 1.63 (Figure 1a). The highest and most significant changes in *HvFPG* expression were measured at 15 h on the 11.48 level. In consecutive hours, the expression was reduced to 0.99 at 24 h. The expression levels of *HvOGG1* between 0.5 h and 12 h were in the range of 1.04 to 3.08 (Figure 1b). The highest level of *HvOGG1* expression was measured at 15 h with 32.39, and in consecutive hours, the expression was reduced to 0.38 at 24 h. The correlation matrix performed for consecutive time points and their biological replicates indicate a strong correlation (r = 0.91, N = 30, *p* < 0.000) between *HvFPG* and *HvOGG1* expression.

Analysis of wheat germinated embryos allowed the determination of *TaFPG* and *TaOGG1* expression. *TaFPG* expressions were determined in the range of 0.37 to 3.0 (Figure 1c). The lowest expression was determined at 15 h and the highest at 9 h. The *TaOGG1* expression was greater in the early hours of germination with a higher level of 3.9 at 9 h (Figure 1d). After this time, expressions were reduced and measured in the range of 0.24 to 0.38. 

Analysis of rye embryos in the same germination conditions showed that the expression of *ScOGG1* is greater than *ScFPG*. At the first two time points, the expression of *ScFPG1* was determined to be 1.3 and 2.0 at 0.5 h and 1 h, respectively (Figure 1e). The highest level of *ScFPG* at the 23.0 level was measured at 3 h, and then reduced in consecutive hours to range from 3.7 to 10.6. Similarity to *ScFPG,* the expression of *ScOGG1* was lowest at 0.5 h and 1 h at the level of 1.5 and 11.6, respectively (Figure 1f). The highest expression level of the *ScOGG1* gene was 116.0 and was measured at 3 h and reduced in consecutive time points to range from 23.6 to 70.1 between 6 h and 24 h. The correlation matrix performed for consecutive time points and their biological replicates indicated a correlation (r = 0.64, N = 30, *p* < 0.000) between *HvFPG* and *HvOGG1* expression.

### 2.2. Species-Dependent Activity of FPG and OGG1 Genes

**Figure 2:**
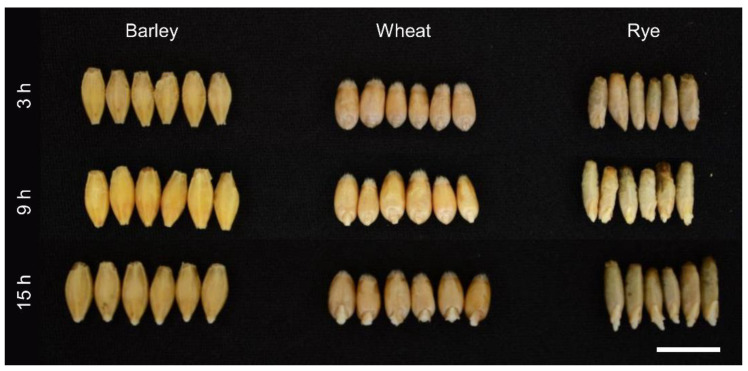
Phenotypic changes in 3-hour (h), 9 h, and 15 h grains of barley, wheat, and rye. Representative example of plant phenotype in six biological replicates, scale bar = 1 cm.

A two-way ANOVA indicates that the expression of *FPG* and *OGG1* genes depends on species and germination time (Table 1). The highest expression of both genes in the rye can be the result of a thin seed coat and greater access to water (Figure 2 and Figure S1). The rye embryos at 3 h from the start of the water treatment had broken seed coats and visible embryos. A similar stage in wheat is determined at 9 h. On the other hand, barley with a seed coat associated with palea and lemma had visible embryos at 15 h.

## 3. Discussion

The base excision repair (BER) system is involved in repairing oxidative damage to deoxyribonucleic acid (DNA). Oxidative stress-induced DNA damage is believed to significantly affect germination and seed viability. Reactive oxygen species (ROS) causes damage to DNA nucleotides by oxidation of sugar residues and strand breakage. The most common ROS-induced damage is guanine modification leading to the formation of 8-oxoguanine (8-oxoG), which causes the GC–TA mutation [26]. Studies on arabidopsis (*Arabidopsis thaliana* L.), barrel medic (*Medicago trancatula* Gaertn.), and sal tree (*Shorea robusta* Gaertn.) have proven that increased levels of 8-oxoG have a negative effect on germination and accelerated seed aging [3,27,28]. DNA damage have negatively affected the integrity of the genome and, if not repaired, can occur to a series of mutations and even cell death. Therefore, proper functioning of repair mechanisms is extremely important for prolonging seed viability and maintaining germination at high levels. Research on arabidopsis has shown that silencing of the DNA repair system in seeds leads to impaired germination [29]. This proves that damage elimination is essential to maintain seed longevity. Oxidative damage caused by ROS in particular 8-oxoG is repaired by the BER system through the participation of specific enzymes such as FPG DNA glycosylase formamidopyrimidime and OGG1 DNA glycosylase 8-oxyguanine. These are DNA glycosylases that hydrolytically cleave the glycosidic bond between deoxyribose and the erroneous base, thereby releasing the damaged base and creating an AP site (apyrimidine/apurine) [30]. The FPG family includes a formamidopyrimidine glycosylase that removes 8-oxoG. In contrast, OGG1 is a bifunctional DNA glycosylase that catalyzes the release of 8-oxoG and DNA cleavage at the resulting site [31]. FPG genes have been described in arabidopsis and sugarcane (*Saccharum officinarum* L.) [32,33]. Plant OGG1 was first isolated and characterized in arabidospis [34,35]. FPG and OGG1 enzymes, by removing 8-oxoG, prevent mutations arising from the action of reactive oxygen species [36]. Studies on barrel medic have shown that the *OGG1* and *FPG* genes are involved in seed repair mechanisms during oxidative stress [27]. Subsequent studies on this species have shown increased expression during the first stages of germination, but also during plant establishment [37]. During seed imbibition, there is an accumulation of oxidative damage, which is a factor that promotes germination [38]. As you can see, ROS is not always the enemy of plants, but their accumulation in larger amounts can lead to serious damage. That is why a strong response of genes involved in the BER repair system is already evident at the early germination stage. This research is the first to check the expression of these two genes in cereals during germination. Analyzing the results, we can conclude that the *FPG* and *OGG1* genes interact with each other. Both genes show the highest expression at the same time in the same species. We can conclude that there is an increased accumulation of 8-oxoG at any given time, which these two genes are able to correct. In each of the species, the pattern is repeated; the highest expressions of *FPG* and *OGG1* occur at the same time. However, the time of the highest expression differs depending on the species studied. The conclusion that can be drawn is that the accumulation of ROS varies between species. In addition, it can be concluded that it depends on how quickly the seeds absorb water. In studies on arabidopsis and barrel medic, *OGG1* gene expression was shown to be affected by the rate of water absorption [39]. Therefore, we suspect that expression levels vary depending on the type of seed coat, which is more or less permeable. Both of these conclusions need to be confirmed experimentally for us to be absolutely certain of their validity. The earliest highest expression of *FPG* and *OGG1* genes was observed in rye, followed by wheat and finally barley. It may be related to the aforementioned different degree of imbibition associated with the presence or absence of the seed coat. The highest level of expression is when the seed coat breaks. As shown in previous studies, this is due to the accumulation of ROS, which must be repaired as quickly as possible to maintain germination potential [40]. Seed drying and water absorption are associated with high levels of ROS, therefore OGG1 activity is highest during seed bursting [6]. The regulation of *FPG* and *OGG1* genes could significantly contribute to increasing seed vigor. This is confirmed by studies on *OGG1* in arabidopsis, where overexpression leads to reduced oxidative damage in dry and hydrated seeds [3]. Furthermore, in soybeans (*Glycine max* L.), it has been shown that an increase in *OGG1* expression results in faster acquisition of germination and thus increased seed vigor [41]. The genes we studied could be used as molecular markers to predict seed quality in a very short time. This would allow for better seed evaluation. Many questions arise in our minds about the activity of *FPG* and *OGG1* and how they affect the aging processes of seeds. In the future, we want to check how the level of ROS accumulation affects the activity of these two genes. It would also be interesting to see how different abiotic factors affect the rate of oxidative damage repair mediated by FPG and OGG1. How different abiotic factors affect the rate of oxidative damage repair mediated by these two genes is also an interesting question that would be worth investigating. Finally, genes involved in DNA repairing during germination will be really important for gene bank implementation while storing cereal grains. Barley is one of the cultivar species with long seed viability, however this depends on the humidity of the samples. Grains stored for 45 years in gene bank conditions present different levels of germination at about 86.7% and 2% [42]. Analysis of total RNA showed that both samples had degraded rRNA particles in comparison to the control, however miRNA and mRNA fractions were stable in all samples [43]. On the other hand, storage of rye samples is one of the bigger challenges for gene banks because grains of this species have a short germination time once fully harvested [44,45].

## 4. Conclusions 

In conclusion, our study showed that the expression levels of the *OGG1* and *FPG* genes are species-dependent. This is probably due to the rate of water assimilation, the timing of which is related to the seed coat type. The *FPG* and *OGG1* genes show correlations with each other, hence the conclusion that they can cooperate with each other. Our research is just an introduction to a better understanding of the role of these two genes in the reduction of oxidative damage and the regulation of seed aging processes. This is the first study describing these genes in wheat, barley, and rye.

## 5. Materials and Methods

### 5.1. Plant Material

In experiments, we used three different species of spring cereals: barley (cv. Farmer), wheat (cv. Alibi), and rye (cv. Fobos). The grains grew in Petri dishes (120 × 120 × 17 mm; 60 seeds per dish) with three layers of filter paper and 13 mL of tap water. Next, they were kept at 21 °C for 24 h. Tissue was collected between 10:00 and 12:00 a.m. by dissection of 25 embryos with scutellum from all species. The experiment consisted of 10 time points from 0.5 h to 24 h after imbibition. Each time point was collected in three biological replicates to liquid nitrogen and then stored in −80 °C pending total RNA extraction.

### 5.2. RNA Extraction, cDNA Synthesis and Real-Time PCR Reaction

Total RNA extraction was performed by triturating the embryonic tissue in liquid nitrogen. Then, we used the isolation method with TRI Reagent™ Solution (Invitrogen, Waltham, MA, USA). Genomic DNA was removed using DNase I, RNase free (Thermo Fisher Scientific, Waltham, MA, USA). Purified total RNA was reverse transcribed into cDNA using the Revert Aid cDNA Synthesis Kit (Thermo Fisher Scientific, Waltham, MA, USA). Specific primers for the *FPG* and *OGG1* genes were designed using reference sequences for barley and wheat (Table S2). These starters were optimized for all tested species. Real-Time PCR was performed using the FastStart Essential DNA Green Master kit (Roche Diagnostics GmbH, Mannheim, Germany) and a LightCycler^®^ 96 Thermocycler (Roche, Mannheim, Germany) according to the manufacturer’s protocols. Two reference genes were used as controls: barley ADP-ribosylation factor and glyceraldehyde-3-phosphate dehydrogenase (GAPDH). For each gene, three biological replicates were performed in three technical repeats.

### 5.3. Data Analysis

Statistical analysis was performed using Microsoft Excel Professional Plus 2016 (Microsoft Office, Warsaw, Poland). The graphs were generated using Microsoft Excel Professional Plus 2016 and Microsoft PowerPoint Professional Plus 2016 (Microsoft Office, Warsaw, Poland). Duncan’s test and two-way ANOVA were performed using the Statistica 13.0 (StatSoft, Kraków, Poland).

## Figures and Tables

**Table 1:** Results of two-way ANOVA for the three species of cultivar cereals (barley, wheat, and rye) or hours of grains germination (0.5 h, 1 h, 3 h, 6 h, 9 h, 12 h, 15 h, 18 h, 21 h, and 24 h) calculated for two genes, i.e., *FPG* and *OGG1*. The asterisks indicate significant dependence for *p* < 0.05 (*) and *p* < 0.001 (**).

Gene	Species			Hours		
	Mean Square	F	*p*	Mean Square	F	*p*
*FPG*	332.31 **	21.2811	0.0000	41.41 *	2.6521	0.0117
*OGG1*	15,945.00 **	33.0171	0.0000	1061.81 *	2.1987	0.0344

## Data Availability

Data are contained within the article or Supplementary Materials.

## Supplementary Materials

The following supporting information can be downloaded at: https://www.mdpi.com/article/10.3390/ijms241512354/s1.
