# Supplementary material for: Profiling of Barley, Wheat, and Rye FPG and OGG1 Genes during Grain Germination
Source: Int J Mol Sci. 2023 Aug 2;24(15):12354. doi: 10.3390/ijms241512354 (PMC10418959; doi:10.3390/ijms241512354)
Supplement: Supplementary file 1 [file ijms-24-12354-s001.zip › Figure S1.pdf]

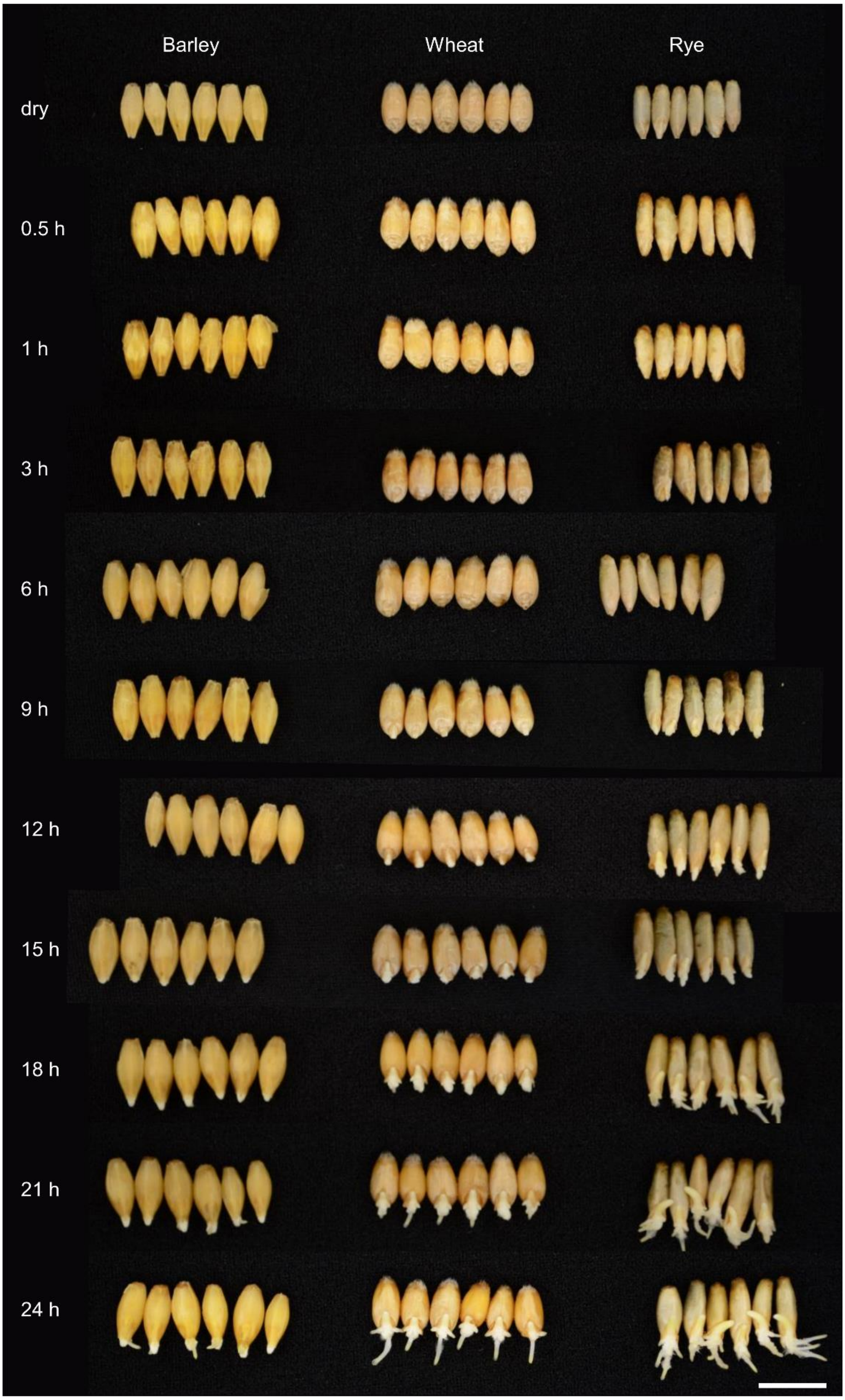

Figure S1. Phenotypic changes in dry, 0.5 hours (h), 1 h, 3, h, 6 h, 9 h, 12 h, 15 h, 18 h, 21 h, and 24 h grains of barley, wheat, and rye. Representative example of plant phenotype in six biological replicates, scale bar = 1 cm.
